# Distinct Clinical Endpoints of *Staphylococcus aureus* Bacteraemia Complicate Assessment of Outcome

**DOI:** 10.1093/cid/ciae281

**Published:** 2024-05-20

**Authors:** Clark D Russell, Karla Berry, George Cooper, Wynne Sim, Rui Shian Lee, Tze Yi Gan, William Donlon, Antonia Besu, Emily Heppenstall, Luke Tysall, Andrew Robb, Simon Dewar, Andrew Smith, Vance G Fowler

**Affiliations:** Centre for Inflammation Research, Institute for Regeneration and Repair, The University of Edinburgh, Edinburgh, United Kingdom; Medical Microbiology, Royal Infirmary of Edinburgh, Edinburgh, United Kingdom; Clinical Infection Research Group, Western General Hospital, Edinburgh, United Kingdom; Centre for Inflammation Research, Institute for Regeneration and Repair, The University of Edinburgh, Edinburgh, United Kingdom; Edinburgh Medical School, The University of Edinburgh, Edinburgh, United Kingdom; Edinburgh Medical School, The University of Edinburgh, Edinburgh, United Kingdom; Edinburgh Medical School, The University of Edinburgh, Edinburgh, United Kingdom; Edinburgh Medical School, The University of Edinburgh, Edinburgh, United Kingdom; Edinburgh Medical School, The University of Edinburgh, Edinburgh, United Kingdom; Medical Microbiology, Royal Infirmary of Edinburgh, Edinburgh, United Kingdom; Medical Microbiology, Royal Infirmary of Edinburgh, Edinburgh, United Kingdom; Scottish Microbiology Reference Laboratory, New Lister Building, Glasgow, United Kingdom; Medical Microbiology, Royal Infirmary of Edinburgh, Edinburgh, United Kingdom; Clinical Infection Research Group, Western General Hospital, Edinburgh, United Kingdom; Scottish Microbiology Reference Laboratory, New Lister Building, Glasgow, United Kingdom; College of Medical, Veterinary & Life Sciences, Glasgow Dental Hospital & School, University of Glasgow, Glasgow, United Kingdom; Division of Infectious Diseases and International Health, Department of Medicine, Duke University School of Medicine, Durham, North Carolina, USA; Duke Clinical Research Institute, Durham, North Carolina, USA

**Keywords:** *Staphylococcus aureus*, bacteraemia, clinical outcomes, mortality, clinical trials

## Abstract

**Background:**

We aimed to test the hypothesis that development of metastatic infection represents a distinct clinical endpoint from death due to *Staphylococcus aureus* bacteremia (SAB).

**Methods:**

We conducted a retrospective observational study of adults with SAB between 20 December 2019 and 23 August st2022 (n = 464). Simple logistic regression, odds ratios, and *z*-scores were used to compare host, clinical, and microbiologic features.

**Results:**

Co-occurrence of attributable mortality and metastatic infection was infrequent. Charlson Comorbidity Index and age were strongly associated with attributable mortality, but not metastatic infection. We compared patients with fatal SAB (without clinically-apparent metastatic complications, 14.4% of cohort), metastatic SAB (without attributable mortality, 22.2%), neither complication (56.7%), and overlapping fatal/metastatic SAB (6.7%). Compared to SAB without complications, fatal SAB was specifically associated with older age and multi-morbidity. Metastatic SAB was specifically associated with community acquisition, persistent fever, persistent bacteremia, and recurrence. Endocarditis was over-represented in the fatal/metastatic SAB overlap group, which shared patient characteristics with fatal SAB. In contrast to other (predominantly musculoskeletal) metastatic complications, endocarditis was associated with increased mortality, with death occurring in older multi-morbid patients later after SAB onset.

**Conclusions:**

Patients with SAB experience distinct clinical endpoints: (i) early death, associated with multi-morbidity and age; (ii) metastatic (predominantly musculoskeletal) SAB; (iii) endocarditis, associated with late death occurring in older people with multi-morbidity, and (iv) bacteraemia without complications. These distinctions could be important for selecting appropriate outcomes in clinical trials: different interventions might be required to reduce mortality versus improve clinical response in patients with metastatic SAB.

## Teaser

We found no overall association between metastatic complications (predominantly musculoskeletal) and death due to *Staphylococcus aureus* bacteraemia. Each endpoint was associated with distinct host, clinical and microbiologic features. Age and multi-morbidity were associated with death but not metastatic infection.

*Staphylococcus aureus* is one of the most common causes of both community-acquired and nosocomial bacteraemia [1]. Among high-incidence bacteremia-causing pathogens, *S. aureus* is associated with the highest mortality [1]. A major distinction in the outcome of patients with SAB is the presence or absence of suppurative metastatic complications, that is, foci of infection which are anatomically distinct from the portal of entry. These are identified in up to 40% of patients [2, 3].

Strategy trials have focussed on combination therapy, especially for methicillin-resistant *S. aureus* bacteraemia (SAB) but to date have not identified approaches that reduce mortality compared to standards of care [4]. Mortality is the endpoint of SAB most commonly captured as an outcome in clinical trials, whereas microbiological and clinical endpoints are used more variably and defined less consistently [4].

After progression of *S. aureus* infection to bacteraemia, metastatic disease and/or attributable mortality are potential clinical endpoints. The extent to which they co-occur or share risk factors is not well described but could be important for assessing the impact of therapeutic interventions in clinical trials. We aimed to test the hypothesis that the development of metastatic infection represents a distinct clinical endpoint from death due to the infection.

## METHODS

### Study Design and Setting

We conducted a retrospective observational study of consecutive adults with SAB at our institution (NHS Lothian, Edinburgh, United Kingdom) between 20 December 2019 and 23 August 2022. Our institution consists of 3 teaching hospitals each with an intensive care unit, together providing tertiary services including infectious diseases, kidney/islet cell/liver transplantation, hematology/oncology, trauma, vascular surgery, neurosurgery, and cardiothoracic surgery. Patients were identified by searching our laboratory information management system to identify all blood cultures with growth of *S. aureus*. The South East Scotland Research Ethics Committee 02 provided a favorable ethical opinion (23/SS/0025).

### Participants

We included unique adults (≥18 years) with SAB. People were excluded if: bacteremia was polymicrobial (except coagulase-negative staphylococci judged to represent contamination); growth of *S. aureus* was considered by an infection specialist to represent contamination; or if no electronic records were available (Supplementary Figure 1). If multiple positive blood cultures were identified for an individual, the first positive was considered to be the index blood culture.

### Variables and Definitions

Comorbidities were defined according to the Charlson Comorbidity Index [5]. Vital signs and laboratory variables were recorded within 24 hours of the index blood culture. Infection was categorized as community-acquired non-healthcare associated, community-acquired healthcare-associated, or nosocomial, according to definitions used by Friedman et al [6]. Source of infection was the most likely portal of entry of *S. aureus* into the bloodstream. A complicated source was a local complication at/contiguous with the portal of entry eg abscess complicating skin and soft tissue infection (SSTI), infected deep vein thrombosis at site of injection drug use, and prosthetic joint infection (PJI) contiguous with a surgical site infection. A metastatic complication was a focus of infection remote from the portal of entry, thought to have arisen through haematogenous dissemination, that was clinically apparent during the index SAB episode. Positive blood cultures following the index blood culture were recorded as (i) positive follow-up blood cultures (if taken 48–96 hours after index blood culture), (ii) persistent bacteraemia (>96 hours after index blood culture and before stopping treatment), or (iii) recurrent bacteremia (within 90 days after stopping treatment and same *S. aureus spa* clonal complex). Attributable in-hospital mortality was determined by review of death certificate and evidence of non-resolving signs/symptoms or foci of SAB at the time of death, and absence of another explanation. *Spa* typing, PVL gene quantitative real-time polymerase chain reaction (qRT-PCR), and confirmation of methicillin-resistant *S. aureus* (MRSA) phenotype by *mecA* qRT-PCR were performed routinely on all blood culture *S. aureus* isolates by the Scottish Microbiology Reference Laboratory.

### Statistical Analysis

Data distributions were assessed using the Shapiro-Wilk test and found to be non-parametric. The Kruskal-Wallis test (multiple groups, with Dunn's multiple comparisons test) or Mann-Whitney test (2 groups) were used as appropriate. Categorical variables were compared by Fisher exact test or the χ^2^ test. Correlation and hierarchical clustering analysis was performed using the *heatmaply* package [7] in R (RStudio version 2023.06.1 + 524). Area-proportional Venn diagrams were produced using the *eulerr* package [8] in R. Relationships between continuous variables were assessed by simple linear regression. The relationship between continuous variables and SAB outcome was assessed by simple logistic regression, and by calculation of odds ratios for categorical variables. To compare variables between SAB outcome sub-groups, *z*-scores were calculated ($z=\frac{\left(value\;for\;subgroup\;-mean\;for\;variable\right)}{standard\;deviation\;for\;variable}$). Survival was visualized by comparison of Kaplan Meier curves using the log-rank test. Unless otherwise stated, analyses and data visualisation were done using GraphPad Prism Version 10.0.3 for macOS.

## RESULTS

### Description of Cohort

We studied 464 eligible adults with predominantly methicillin-susceptible *S. aureus* bacteremia (447/464, 96.3%; Table 1). Participants had a median age of 68 years (interquartile range [IQR] 52–79 years), and 294 (63.4%) were male. The median Charlson Comorbidity Index of participants was 4 (IQR 2–7), with diabetes mellitus the most common individual comorbidity. SAB was community acquired nonhealthcare-associated in 181 cases (39.0%). The source of SAB was unknown in 170 cases (36.6%), an intravenous catheter in 93 (20.0%), and SSTI in 88 (19.0%). During the index SAB episode, 134 participants had clinically apparent metastatic foci (28.9%), and 98 suffered attributable in-hospital mortality (21.1%).

**Table 1.** Cohort Characteristics

	Number of Patients (%)
Age, y (median (IQR))	68 (52–79)
Male sex	294 (63.4)
Body mass index, kg/m^2^, median (IQR)^a^	26.1 (22.5–30.7)
Comorbidities	
** **Charlson Comorbidity Index, median (IQR)	4 (2–7)
** **Diabetes mellitus (all)	120 (25.9)
** **Heart failure	78 (16.8)
** **Stroke	75 (16.2)
** **Diabetes mellitus with complications	66 (14.2)
** **Myocardial infarction	58 (12.5)
** **Chronic obstructive pulmonary disease	48 (10.3)
** **Localized cancer	47 (10.1)
** **Dementia	45 (9.7)
** **Chronic kidney disease	35 (7.5)
** **Connective tissue disease	35 (7.5)
** **Metastatic cancer	33 (7.1)
** **Peripheral vascular disease	32 (6.9)
** **Moderate-severe liver disease	26 (5.6)
** **Hemodialysis	18 (3.9)
Person who injects drugs	41 (8.8)
Surgery in 30d prior to index blood culture	51 (11.0)
Presence of implantable prosthetic material	
** **Any type	182 (39.2)
** **Central venous catheter (any type)	49 (10.6)
** **Vascular graft	21 (4.5)
** **Implantable cardiac device^b^	24 (5.2)
** **Prosthetic heart valve	24 (5.2)
** **Orthopaedic implant	91 (19.6)
Site of acquisition	
** **Community-acquired non-healthcare-associated	181 (39.0)
** **Community-acquired healthcare-associated	113 (24.4)
** **Nosocomial	170 (36.6)
Source of bacteraemia	
** **Unknown	170 (36.6)
** **Intravenous catheter (any kind)	93 (20.0)
** **Skin or soft tissue infection^c^	88 (19.0)
** **Injection drug use	33 (7.1)
** **Urinary^d^	25 (5.4)
** **Bone^e^	27 (5.8)
** **Respiratory	28 (6.0)
Complicated source^f^	64 (13.8)
Any metastatic complication	134 (28.9)
Microbiology	
** **Methicillin-resistant *S. aureus*	17 (3.7)
** **PVL toxin genes detected	4 (0.9)
** **Positive follow-up blood culture^g^	23/314 (7.3)
** **Persistent bacteraemia^h^	7 (1.5)
Outcomes	
** **Recurrent bacteraemia^i^	9 (1.9)
** **In-hospital attributable mortality	98 (21.1)
** **In-hospital all-cause mortality	110 (23.7)
** **90-day all-cause mortality	130 (28.0)

Abbreviations: BMI, body mass index; IQR, interquartile range; PVL, Panton-Valentine Leucocidin; *S. aureus*, *Staphylococcus aureus*.

^a^109/449 (24.3%) patients with data available had a BMI >30 kg/m^2^.

^b^Implantable pacemakers, automated implantable cardioverter-defibrillators, and left ventricular assist devices.

^c^Including surgical site infections.

^d^Including urinary catheter-associated urinary tract infections.

^e^For example, osteomyelitis underlying an infected ulcer (if both bone and skin or soft tissue were present then the source was recorded as bone).

^f^Local complication at/contiguous with portal of entry eg abscess, osteomyelitis, thrombophlebitis, prosthetic joint infection.

^g^Expressed as percentage of n = 314 patients with blood cultures repeated 48–96 h after index blood culture.

^h^Positive blood culture >96 h after index blood culture and before stopping treatment.

^i^Positive blood culture ≤90 d after stopping treatment, with *S. aureus* of same clonal complex.

Of the 134 patients with SAB and clinically apparent metastatic foci, the median number of identified foci was 2 (IQR 1–2; range 1–5). Vertebral osteomyelitis (n = 54, 11.6%) was most common, followed by endocarditis (n = 35, 7.5%), then native joint septic arthritis (n = 29, 6.3%; Supplementary Figure 2*A*). Endocarditis fulfilled the modified Duke criteria as “definite” in 28 and “possible” in 7 cases [9]. Eight cases involved a prosthetic heart valve and 29 involved left-sided valves (native or prosthetic). Overall, musculoskeletal metastatic complications were most common (Supplementary Figure 2*B*). Correlation analysis of metastatic complications present in individual patients identified clusters of co-occurring complications (Supplementary Figure 2*C*). Endocarditis with emboli (brain, spleen, and kidney) represented an entirely separate cluster from other predominantly musculoskeletal complications.

### Limited Overlap in Components of Complicated SAB

**Figure 1.**
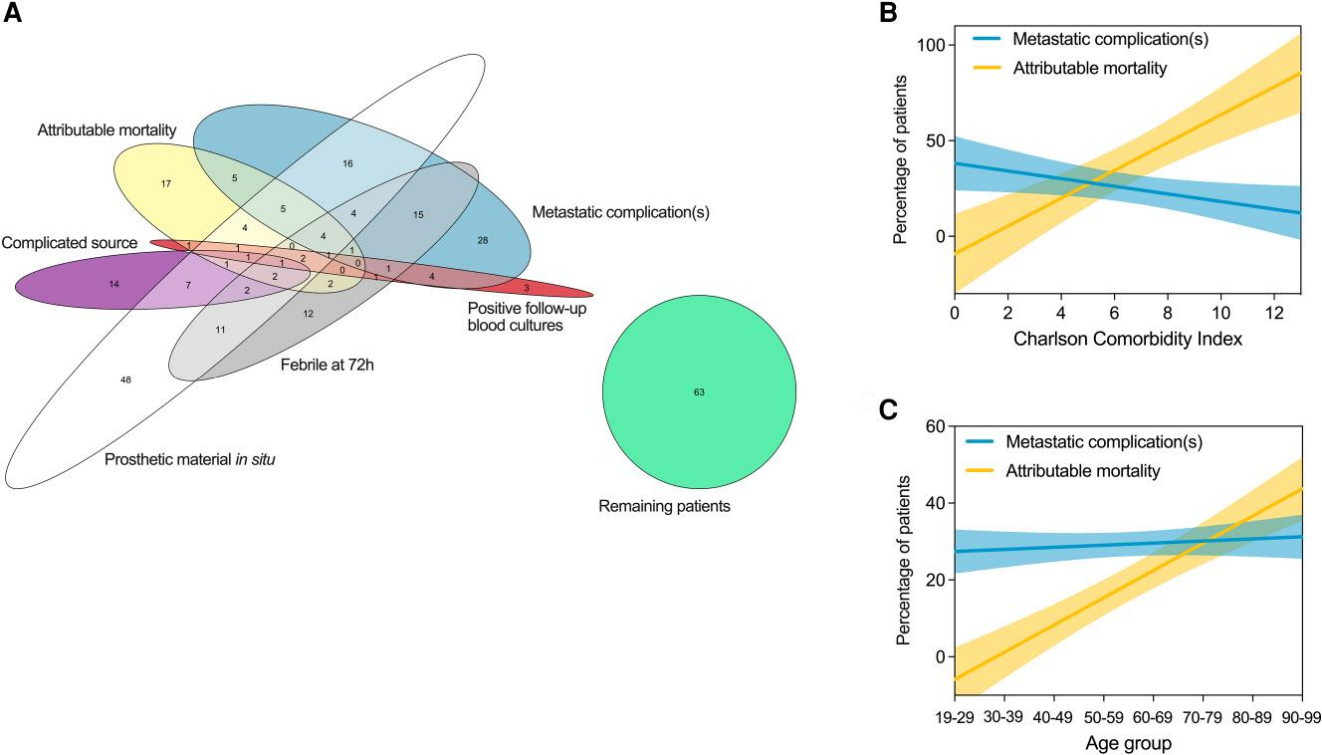
Components and patient characteristics of complicated SAB. *A*, Overlap in components of “complicated” SAB (IDSA and Fowler et al) co-occurring in the same patients. Numbers indicate the number of patients. This analysis was restricted to 290 patients with data recorded for follow-up blood cultures (performed within 48–96 h of index blood culture) and temperature on day 3 after index blood culture. Simple linear regression of (*B*) Charlson Comorbidity Index and (*C*) age with metastatic complications and attributable in-hospital mortality. Lines show regression lines and shaded areas show 95% confidence intervals. Abbreviations: IDSA, Infectious Diseases Society of America; SAB, *Staphylococcus aureus* bacteremia.

In total. 262 people (56.4%) met the definition of complicated SAB used by the IDSA [10]. When a definition of complicated SAB used by Fowler et al was applied, 254 (54.7%) met the definition [11] with an overlap of 189 with the IDSA definition. These figures could be an under-estimate as follow-up blood cultures were not available for 150 patients (32.3%). There was limited overlap in co-occurrence of the individual components of complicated SAB in the same patients (Figure 1*A*). In particular, the occurrence of metastatic complications and attributable mortality were largely discrete. The presence of a metastatic complication was not associated with increased attributable in-hospital or all-cause 90-day mortality in this cohort (Supplementary Figure 3).

We then compared the relationship of Charlson Comorbidity Index and age with metastatic complications and with attributable mortality (Figure 1*B*). Charlson Comorbidity Index was strongly positively associated with attributable mortality (slope +7.3, standard error [SE] 1.2, *P* < .0001). In contrast, Charlson Comorbidity Index was negatively associated with metastatic complications, with a flatter slope (slope −2.0, SE 0.8, *P* = .04). Similarly, age (Figure 1*C*) was strongly positively associated with attributable mortality (slope +7.1, SE 0.8, *P* = .0001) but not with metastatic complications (slope +0.6, SE 0.6, *P* = .4). Considering the major clusters of metastatic complications separately, people with endocarditis, vertebral osteomyelitis, and septic arthritis and/or metastatic abscesses all had lower age and Charlson Comorbidity Index compared to people without metastatic complications who suffered attributable mortality (Supplementary Figure 4).

### Host, Clinical and Microbiologic Features Differentiate Metastatic and Fatal SAB

We then aimed to determine if host, clinical, or microbiologic characteristics were specifically associated with metastatic SAB compared to fatal SAB. To do this we partitioned the cohort into 4 groups (Supplementary Figure 5*A*): people with clinically apparent metastatic complications during index SAB episode who did not suffer attributable in-hospital mortality (“mSAB”; 22.2% of cohort); people who did suffer attributable in-hospital mortality and did not have clinically-apparent metastatic complications (“fSAB”; 14.4%); people without metastatic complications during index SAB episode who did not suffer attributable mortality (SAB without complications; 56.7%); and people with both metastatic complications and who suffered attributable mortality during index SAB episode (“overlap”; 6.7%). Consistent with the entire cohort, vertebral osteomyelitis remained the most common metastatic complication in the mSAB group but endocarditis was over-represented in the fatal/metastatic overlap group (16/31 patients had endocarditis compared to 19/103, *P* = .0008; Supplementary Figure 5*B*).

**Figure 2.**
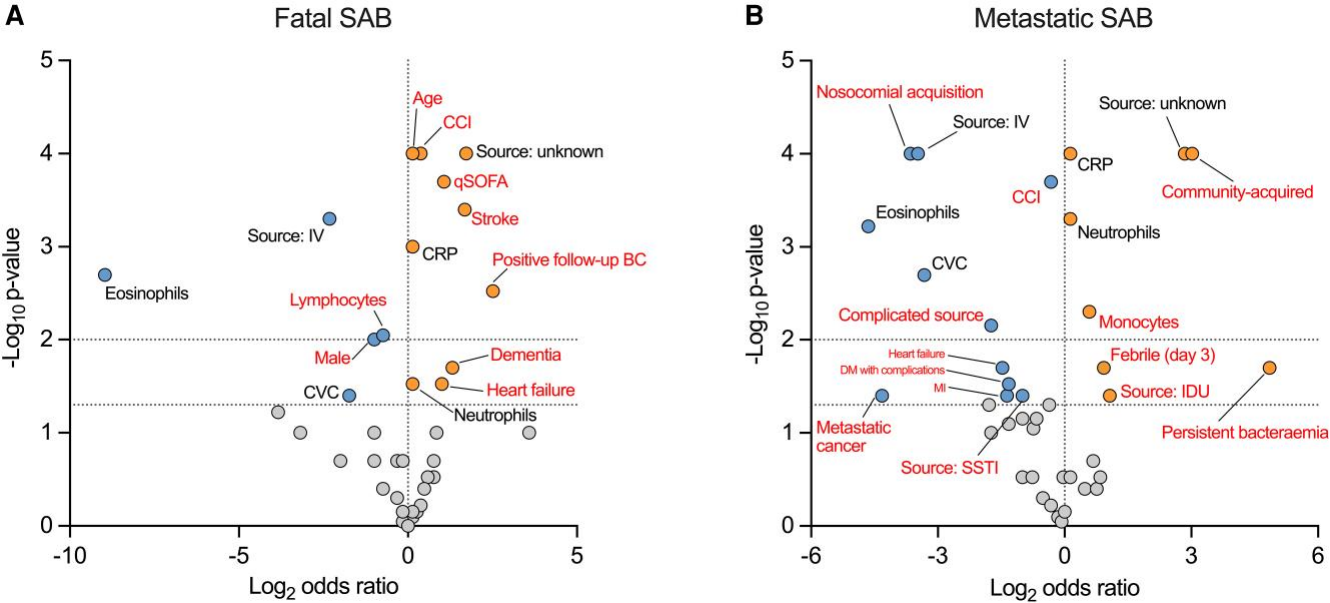
Host, clinical, and microbiologic features of metastatic and fatal SAB. Features associated with (*A*) fatal SAB and (*B*) metastatic SAB compared to the group of patients with neither complication during index SAB episode. Volcano plot of log_2_ odds ratio. Horizontal dotted lines indicate *P* = .05 and *P* = .01. Features are colored based on effect size and statistical significance (*P* < .05): positive association (orange), negative association (blue), or no association (grey). Features labelled in red font are specific to either metastatic or fatal SAB. Abbreviations: BC, blood culture; CCI, Charlson Comorbidity Index; CRP, C-reactive protein; CVC, central venous catheter; DM, diabetes mellitus; qSOFA, quick Sequential Organ Failure Assessment score; IDU, injection drug use; IV, intravenous; MI, myocardial infarction; PWID, person who injects drugs; SAB, *Staphylococcus aureus* bacteremia; SSTI, skin or soft tissue infection.

Characteristics of the mSAB and fSAB groups were compared to the SAB without complications group (Figure 2*A* and *B*, Supplementary Figure 5*C*–*F*), identifying specific features associated with each endpoint, in addition to features common to both. Comorbidity, age, acute illness severity (qSOFA score), and positive follow-up blood cultures (48–96 hours after index) were specifically positively associated with fSAB. Lymphocyte counts and male sex were specifically negatively associated with fSAB. In contrast, non-healthcare-associated community-acquisition, bacteraemia originating from injection drug use, monocyte counts, persistent fever (≥38°C on day 3 after index blood culture) and persistent bacteraemia (positive blood culture >96 hours after index but before stopping treatment) were specifically positively associated with mSAB. Metastatic cancer, Charlson Comorbidity Index, SSTI source of bacteraemia, and complicated infection source were specifically negatively associated. A stepwise increase in baseline CRP was observed from the SAB without complications group to fSAB to mSAB (Supplementary Figure 5*F*). The distribution of *spa* clonal complex did not differ between the groups, although CC030 was numerically more prevalent in the small fatal/metastatic overlap group, consistent with other reports [12, 13] (Supplementary Figure 5*G*). There was also no difference in the distribution of *spa* clonal complex when patients were stratified by presence/absence of endocarditis or vertebral osteomyelitis (Supplementary Figure 6).

We were concerned about the risk of immortal time bias in this analysis. It is likely some people died prior to the diagnosis of a metastatic complication that was truly present so could be wrongly assigned to the fSAB group. Potentially consistent with this was the difference in time to attributable mortality between the fSAB group (median 7 days, IQR 3–16) and the fatal/metastatic overlap group (median 25 days, IQR 15–37). In an effort to address this we compared the variables identified above between the mSAB, fSAB, SAB without complications, and overlap groups, and sub-groups of fSAB, split by time to death (≤7 days and >7 days) (Supplementary Figure 7). The early and late sub-groups of fSAB had very similar characteristics, remaining distinct from mSAB, suggesting the differences observed compared to mSAB were not exclusively an artefact of immortal time bias.

Recurrent SAB was identified in 9 cases (1.9%), a median of 17d (IQR 6.5–51.5) after stopping treatment for the index episode. One recurrence in the mSAB group was associated with an additional metastatic focus (new psoas abscess), and 2 had unresolved previously apparent metastatic foci. All 3 recurrences in the group with SAB without complications during the index episode had metastatic complications newly apparent at the time of recurrence (endocarditis, n = 2; prosthetic joint infection, n = 1).

### Endocarditis is a Separate Endpoint From Other Metastatic Complications of SAB

Age, comorbidity, and qSOFA score all remained more similar between the fSAB and fatal/metastatic overlap groups compared to mSAB and SAB without complications (Supplementary Figure 5*C*–*F*). Unknown source of bacteraemia and CRP were similar between the mSAB and overlap groups. However, patients in the overlap group were more likely to have community-acquired healthcare-associated SAB (Supplementary Table 1).

**Figure 3.**
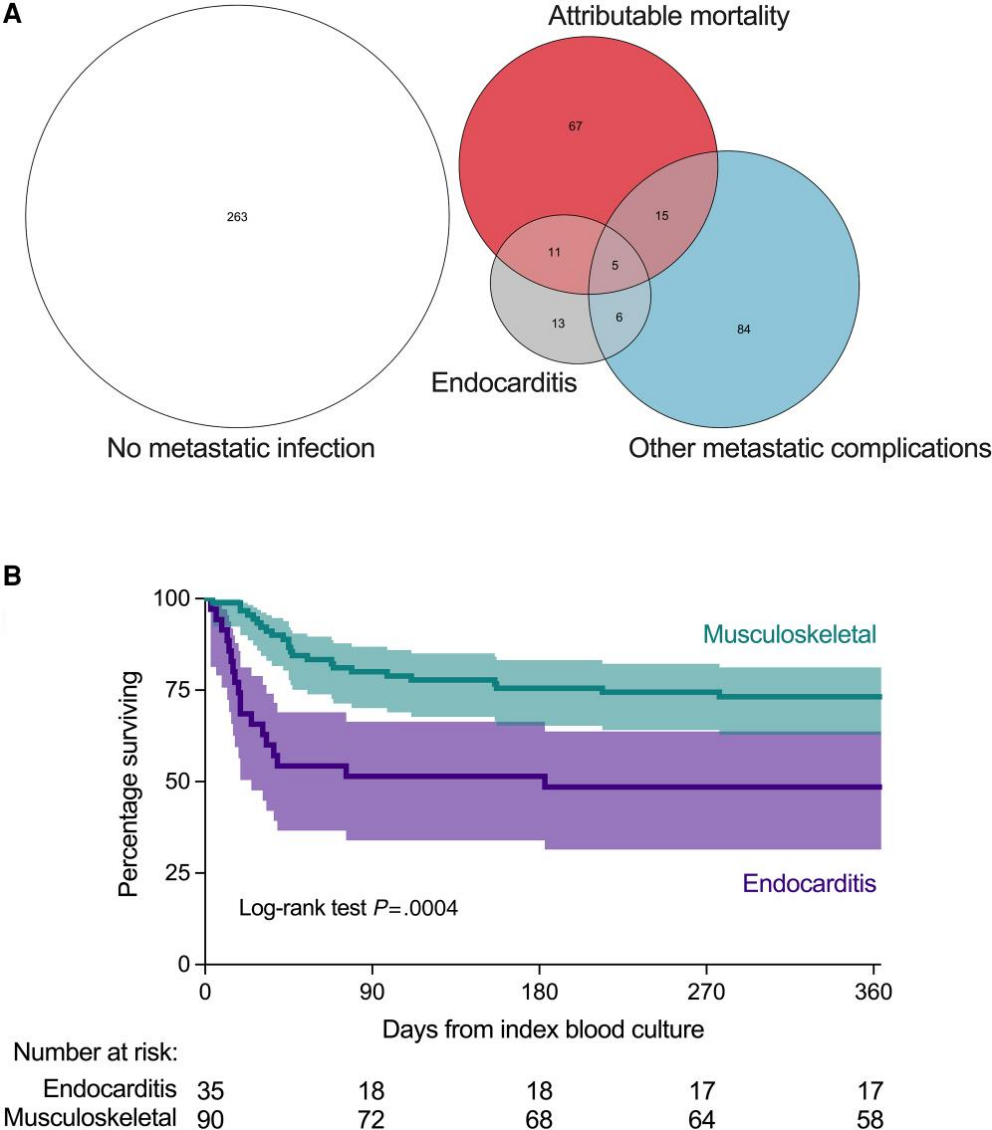
Distinct clinical endpoints of SAB. *A*, Overlap between progression to attributable in-hospital mortality, endocarditis, other metastatic complications, or none of these complications during the index SAB episode. Overlap between sets indicates patients experienced all of the overlapping endpoints, for example, attributable mortality and endocarditis. Numbers indicate the number of patients. *B*, Unadjusted 365-day survival. Kaplan-Meier survival curve, shaded area represents 95% confidence interval for percentage surviving. Musculoskeletal metastatic complications were vertebral osteomyelitis, septic arthritis (native or prosthetic joint), and deep muscle abscesses. Abbreviation: SAB, *Staphylococcus aureus* bacteremia.

Endocarditis was the most common metastatic complication in the overlap group compared to the mSAB group (Figure 3*A*). Of the total of 35 patients diagnosed with endocarditis during the index SAB episode, 16 (45.7%) suffered attributable in-hospital mortality (after a median of 17 days, IQR 12–30). In contrast to patients with metastatic complications of any type, endocarditis was associated with increased attributable mortality compared to the remainder of the cohort (45.7% vs 19.1%, *P* = .0008). Attributable mortality was higher among people with endocarditis compared to people with vertebral osteomyelitis or septic arthritis and/or metastatic abscesses (Supplementary Figure 8). All-cause mortality from index blood culture to 365 days was compared between patients with endocarditis and musculoskeletal metastatic complications, confirming a significant difference, due to early mortality in people with endocarditis (Figure 3*B*).

Patients with endocarditis in the mSAB (n = 19) and overlap (n = 16) groups were compared (ie, non-fatal vs fatal endocarditis), identifying that fatal *S. aureus* endocarditis was associated with older age (median 75 years [IQR 59–82] vs 68 [48–76], *P* = .06) and increased Charlson Comorbidity Index (median 5 [3.5–8.0] vs 2 [1–4], *P* = .008). Most non-fatal endocarditis was non-healthcare-associated community-acquired (14/19), whereas most fatal endocarditis was nosocomial or healthcare-associated community-acquired (11/16; *P* = .04). Bacteremia was from an unknown source in the majority of cases in both groups (12/19 and 11/16). Overall, compared to musculoskeletal metastatic complications, development of endocarditis represents a separate endpoint associated with death in a sub-group of older patients with multi-morbidity.

## DISCUSSION

In this UK cohort of predominantly MSSA bacteraemia, metastatic infection (predominantly musculoskeletal) and attributable mortality represented frequent and distinct complications. Progression to early death (median 7 days from SAB onset) was specifically associated with older age, multi-morbidity, and increased acute illness severity, but these features were not associated with metastatic SAB. A small sub-group of patients with metastatic SAB did suffer attributable mortality, and this was associated with endocarditis. SAB with endocarditis was associated with later death (median 17 days), occurring in older people with multi-morbidity, identifying endocarditis as a further distinct but less frequent endpoint of SAB.

Predictors of death due to SAB have been extensively investigated. Age, comorbidity, and illness severity are reproducibly associated with death [14, 15], and this has been recognized since the earliest observational studies of this disease [16].

Fewer studies have investigated features specifically associated with metastatic SAB. In the largest existing prospective study of SAB, conducted in the United States, the MRSA USA300 clone, male sex, diabetes mellitus, corticosteroid use, and non-healthcare-associated community-acquired SAB were positively associated with metastatic complications [2]. Neoplasia, solid organ transplantation, and SAB from a CVC source were negatively associated [2]. Direct comparison with this cohort is confounded by differences in cohort characteristics, including MRSA bacteraemia (3.7% vs 48.0%) and prevalence of diabetes mellitus (26.0% vs 38.6%). No USA300 isolates were identified in the current study (ie, PVL-positive MRSA *spa* CC008). Furthermore, the distribution of metastatic complications differed between these cohorts, with the current study finding more vertebral osteomyelitis (11.6% vs 4.5%) and less endocarditis (7.5% vs 13.7%). In a Japanese cohort with MSSA bacteraemia (excluding people who died), community-acquired bacteremia, unknown source of bacteremia, persistent fever (>72 hours), delayed treatment, and CRP concentration were positively associated with metastatic complications [17]. SAB from a CVC source was negatively associated [17]. In a prospective study of time to blood culture positivity in a cohort of 357 patients in the United States (52% MRSA), metastatic SAB was more likely if time to blood culture positivity was ≤14 hours [18]. Other predictors of metastatic SAB were not sought. In a study specifically investigating CVC-associated SAB, predictors of metastatic complications were increased symptom duration, haemodialysis, presence of prosthetic material, and MRSA [19]. Despite cohort differences, consistent features of metastatic SAB emerge from 3 of the cohorts, including non-healthcare-associated community acquisition, unknown source, higher CRP, persistent fever, and absence of an association with age or multi-morbidity (Supplementary Figure 9). This provides confidence regarding the generalisability of the current study. Furthermore, although not directly comparable, persistent bacteraemia (this study) and reduced time to positivity [18] both suggest bacterial load is relevant. Community-acquisition (this study) is also likely correlated with increased symptom duration prior to diagnosis, which was identified in the study of CVC-associated SAB [19].

Our study has important limitations. Data collection was retrospective, and the timing of development/diagnosis of metastatic infection was not recorded. The impact of appropriate initial antimicrobial treatment were not assessed. Diagnostic evaluation was at the discretion of the treating clinicians and was not recorded as part of the study (eg, which patients underwent TOE). Positron emission tomography–computed tomography (PET-CT) has the potential to identify metastatic foci that are not clinically apparent [20] but was not used in any case. Overall, there is risk of misclassifying patients as not having metastatic foci when these were truly present but not identified. There was no prospective follow-up of patients, so identification of recurrent SAB was entirely opportunistic, relying on symptoms leading to healthcare attendance and then blood cultures being collected. The potential for immortal time bias in the differentiation of mSAB from fSAB has been discussed, as have differences in the prevalence of endocarditis and vertebral osteomyelitis compared to a large US cohort [2]. The prevalence of diagnosed endocarditis in the current study is similar to that reported in other UK and European cohorts (5.7–10.6%) [21].

We propose that metastatic infection (predominantly musculoskeletal) and early death are distinct endpoints complicating SAB. Endocarditis is a less common but further separate endpoint, associated with later death in the context of multi-morbidity. We suggest these complications be considered separately from a therapeutic perspective: different interventions might be required to reduce mortality compared to improving clinical response in metastatic disease. In patients at risk of progression to fatal SAB, intensification of antimicrobial treatment, for example, with combination therapy, may be less beneficial compared to approaches to modify the host response to prevent immunopathology and decompensation of comorbid conditions. In a retrospective analysis of sepsis-associated deaths, most underlying causes of death related to comorbidities and the majority were not considered preventable by available hospital-based interventions [22]. In contrast, features associated with metastatic SAB (predominantly musculoskeletal) are consistent with a higher bacterial load and/or impaired bacterial clearance. Metastatic SAB may therefore be more amenable to intensified antimicrobial treatment, and aggressive source identification and control (eg, directed by PET-CT). Informative outcome measures may be clinical response and recurrence, not survival. Although susceptibility to fatal SAB was strongly associated with multi-morbidity, susceptibility to metastatic SAB was less well explained. Host factors predisposing to metastatic SAB warrant further investigation as they could represent treatable traits [23], especially if defective microbicidal responses are implicated that could be augmented by host-directed therapies. Finally, patients not at risk of metastatic or fatal SAB may benefit from alternative approaches, such as earlier IV to oral switch [24]. In summary, patients with SAB experience distinct clinical endpoints which complicate the assessment of outcome in SAB and could therefore be important for clinical trial design.

## Supplementary Data

Supplementary materials are available at *Clinical Infectious Diseases* online. Consisting of data provided by the authors to benefit the reader, the posted materials are not copyedited and are the sole responsibility of the authors, so questions or comments should be addressed to the corresponding author.

## Supplementary Material

ciae281_Supplementary_Data

## Notes

***Author Contributions.*** C. D. R. conceived the study. C. D. R., K. B., G. C., W. S., R. S. L., T. Y. G., W. D., A. B., E. H., L. T., and A. R, curated the data. C. D. R. did the investigation. C. D. R. supervised the study. C. D. R. wrote the original draft of the manuscript. V. G. F., S. D., A. S., K. B., and G. C. reviewed and edited the manuscript. C. D. R. validated the study data. C. D. R., K. B., G. C., and E. H. had access to the raw data. The corresponding author had full access to all the data and final responsibility for the decision to submit for publication.

***Acknowledgments.*** This work uses data provided by patients and collected by the National Health Service (NHS) as part of their care.

***Data availability.*** Please contact the corresponding author to discuss access to the dataset. We would welcome opportunities to share data and contribute to collaborative analyses.

***Financial support*.** V. G. F. was supported by grant number R01-AI165671 from the National Institutes of Health. C. D. R. was supported by an Edinburgh Clinical Academic Track (ECAT)/Wellcome Trust PhD Training Fellowship for Clinicians award (214178/Z/18/Z).
