# Supplementary material for: Distinct Clinical Endpoints of Staphylococcus aureus Bacteraemia Complicate Assessment of Outcome
Source: Clin Infect Dis. 2024 May 20;79(3):604–11. doi: 10.1093/cid/ciae281 (PMC11426269; doi:10.1093/cid/ciae281)
Supplement: ciae281_Supplementary_Data [file ciae281_supplementary_data.pdf]

Distinct clinical endpoints of *Staphylococcus aureus* bacteraemia complicate assessment of outcome

Supplementary Material

Contents:

Supplementary Table 1: Characteristics of SAB endpoint groups 2

Supplementary Figure 1: Flow diagram of study cohort selection... 3

Supplementary Figure 2: Clusters of co-occurring metastatic complications. 4

Supplementary Figure 3: SAB mortality stratified by presence/absence of metastatic complications. 5

Supplementary Figure 4: Age and Charlson Comorbidity Index stratified by SAB complication. 6

Supplementary Figure 5: Host, clinical and microbiologic features of metastatic and fatal SAB. 7

Supplementary Figure 6: Clonal complex distributions in vertebral osteomyelitis and infective endocarditis...... 8

Supplementary Figure 7: Comparison of discriminatory features between SAB endpoint groups. 9

Supplementary Figure 8: Attributable in-hospital mortality stratified by metastatic complications. 10

Supplementary Figure 9: Overlap in features of metastatic SAB between studies. 11

Supplementary Table 1: Characteristics of SAB endpoint groups

	**Metastatic** (n=103)	**Fatal** (n=67)	**Without complications** (n=263)	**Overlap** (n=31)
Age, years (median (IQR))	64 (48-77)	77 (69-85)	63 (48-74)	78 (73-83)
Male sex	66 (64.1)	33 (49.2)	173 (65.8)	22 (71.0)
Charlson Comorbidity Index (median (IQR))	2 (1-5)	6 (5-9)	4 (2-7)	5 (4-7)
Acquisition				
Community-acquired non-healthcare-associated	78 (75.7)	20 (29.9)	73 (27.8)	10 (32.3)
Community-acquired healthcare-associated	18 (17.5)	16 (23.9)	68 (25.9)	11 (35.5)
Nosocomial	7 (6.8)	31 (46.3)	122 (46.4)	10 (32.3)
qSOFA (median (IQR))	0 (0-1)	1 (1-2)	1 (0-1)	1 (1-2)
Implantable prosthetic material present (any type)	32 (31.1)	26 (38.8)	109 (41.4)	15 (48.4)
Source				
Unknown	66 (64.1)	30 (44.8)	52 (19.8)	21 (67.7)
Intravenous catheter (any kind)	4 (3.9)	5 (7.5)	80 (30.4)	4 (12.9)
Skin or soft tissue infection	13 (12.6)	12 (17.9)	58 (22.1)	5 (16.1)
Injection drug use	14 (13.6)	0 (0)	18 (6.8)	1 (3.2)
Urinary	2 (1.9)	2 (2.9)	21 (8.0)	0 (0)
Bone	2 (1.9)	3 (4.5)	22 (8.4)	0 (0)
Respiratory	1 (1.0)	15 (22.4)	12 (4.6)	0 (0)
Complicated source	7 (6.8)	7 (10.4)	49 (18.6)	1 (3.2)
Positive follow-up blood culture*	5/78 (4.8)	6/34 (17.6)	7/180 (2.7)	5/23 (22.7)
Persistent bacteraemia	5 (4.8)	1 (1.5)	0 (0)	1 (3.2)
Recurrent bacteraemia	4 (3.9)	NA	3 (1.1)	2 (6.5)

Table shows number (%) unless otherwise stated.
*expressed as percentage of patients with blood cultures repeated 48-96h after index blood culture

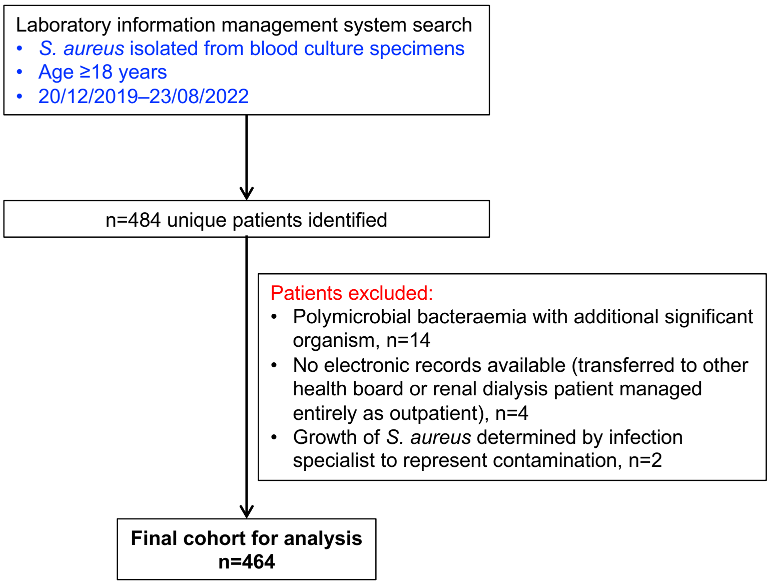

Supplementary Figure 1: Flow diagram of study cohort selection.

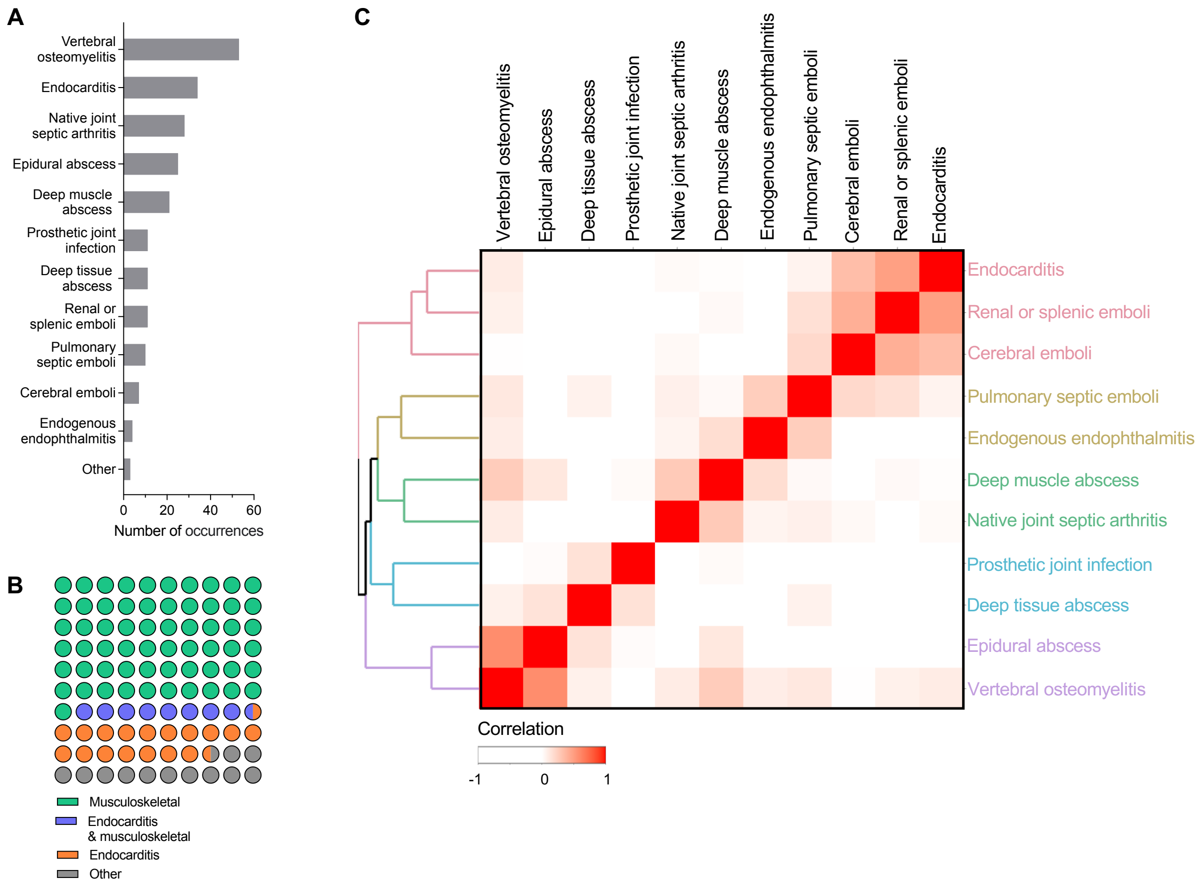

Supplementary Figure 2: Clusters of co-occurring metastatic complications.
(A) Overall frequency of metastatic complications. Deep tissue abscesses involved liver, kidney, prostate, lung or skull base. Deep muscle abscesses involved the psoas, iliacus, paravertebral, gluteal, obturator, or erector spinae muscles. Other metastatic complications were empyema (n=1) and vascular graft infection (n=2). **(B)** Categories of metastatic complication. 'Other' includes septic pulmonary emboli, deep tissue abscesses, empyema, and vascular graft infection. **(C)** Clustered heatmap of the correlation between co-occurring metastatic complications. Dendrogram and coloured text illustrate results of hierarchical clustering of co-occurring complications. Shading of cells represents Pearson's correlation coefficient.

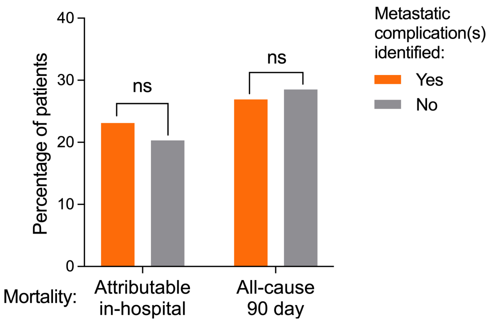

Supplementary Figure 3: SAB mortality stratified by presence/absence of metastatic complications.

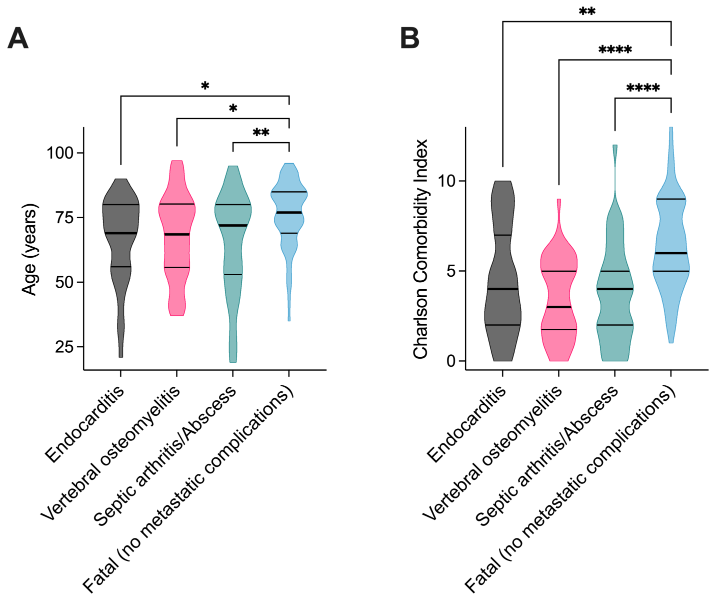

Supplementary Figure 4: Age and Charlson Comorbidity Index stratified by SAB complication. Violin plots, horizontal lines indicate median and interquartile range, outline of plot illustrates the frequency distribution by kernel density estimate. * p<0.05; ** p<0.01; **** p<0.0001 (adjusted for multiple comparisons).

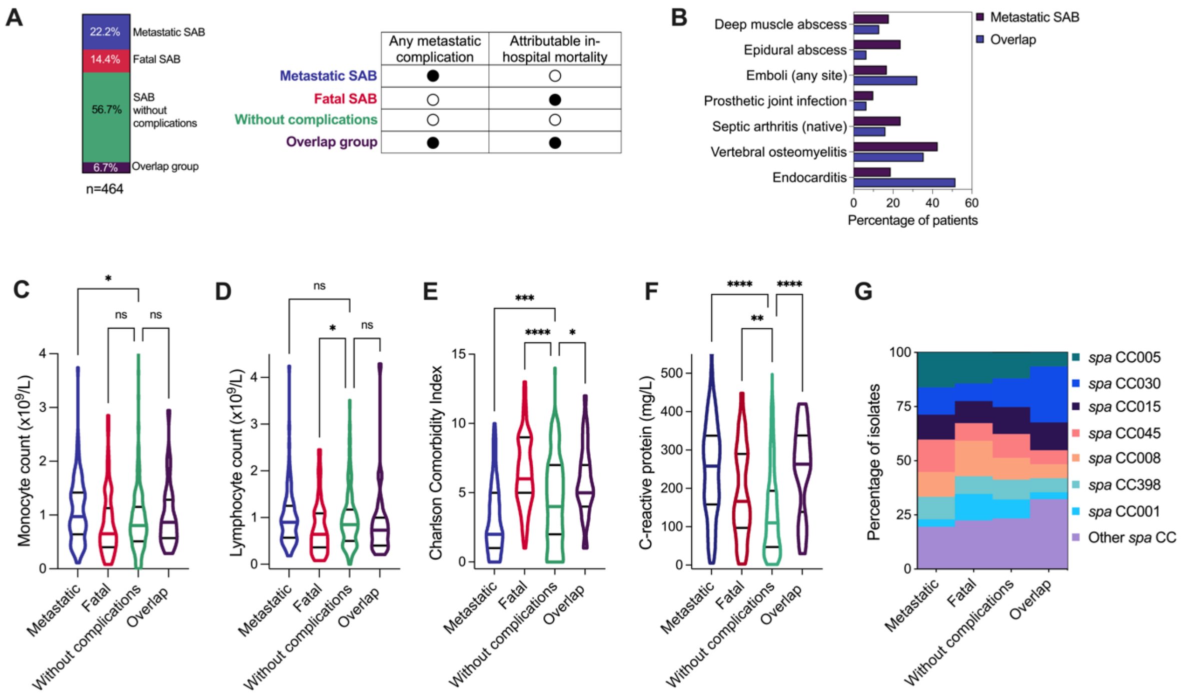

Supplementary Figure 5: Host, clinical and microbiologic features of metastatic and fatal SAB.
(A) The entire cohort was partitioned into four groups to investigate features specifically associated with metastatic and fatal SAB. **(B)** Distribution of metastatic complications in the metastatic SAB group and the overlap group. Distribution of **(C)** monocyte count, **(D)** lymphocyte count, **(E)** Charlson Comorbidity Index, and **(F)** C-reactive protein between the groups. Laboratory values referred to were obtained within 24h of index blood culture. Violin plots, horizontal lines indicate median and interquartile range, outline of plot illustrates the frequency distribution by kernel density estimate. Comparisons are made to the SAB without complications group: * p<0·05; ** p<0·01; *** p<0·001, **** p<0·0001 (adjusted for multiple comparisons). **(G)** Distribution of *S. aureus spa* inferred clonal complexes of bloodstream isolates between groups.

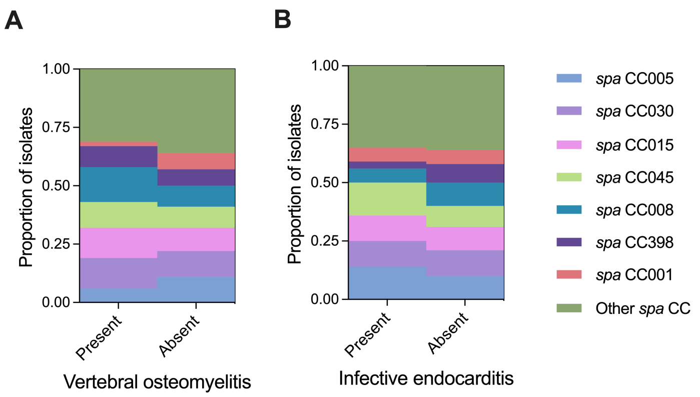

Supplementary Figure 6: Clonal complex distributions in vertebral osteomyelitis and infective endocarditis.

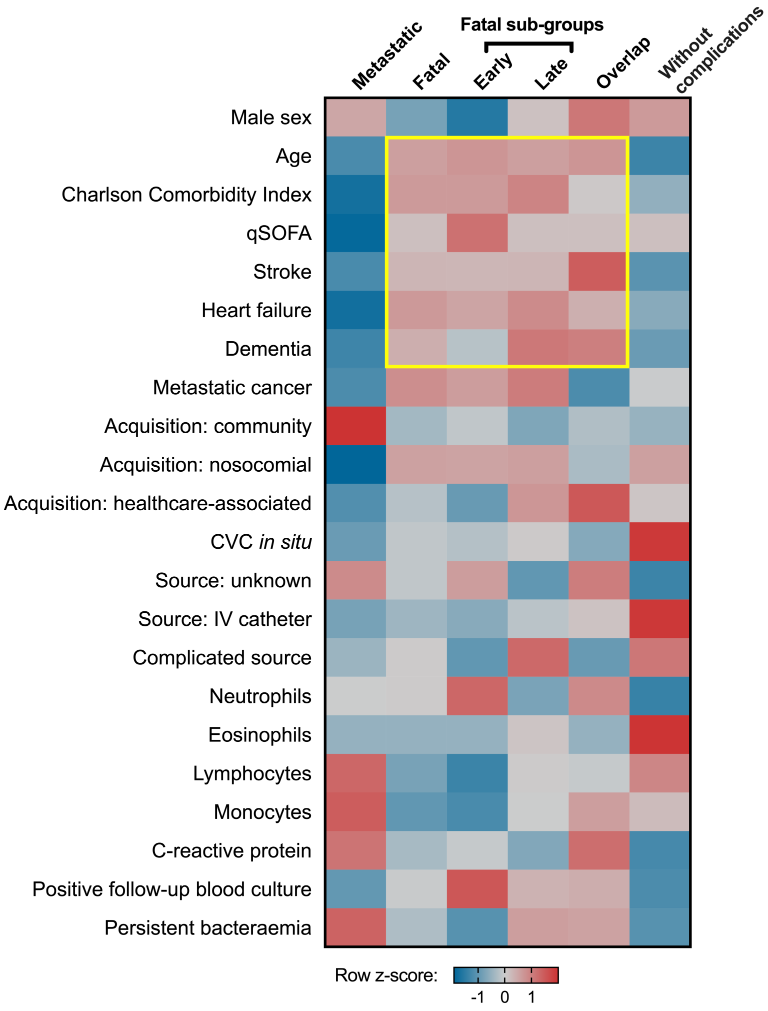

Supplementary Figure 7: Comparison of discriminatory features between SAB endpoint groups. Variation in features between SAB endpoint groups. Patients in the fatal SAB group were sub-divided into early death (attributable mortality ≤7d from index blood culture) and late death (>7d). Cells in the heatmap are shaded by row z-score (determined using percentage for categorical variables and median for continuous variables). Yellow box added to highlight variables discussed in text. Group sizes: metastatic, n=103; fatal, n=67; overlap (attributable mortality and metastatic foci), n=31; early death, n=37; late death, n=30; SAB without complications, n=263.

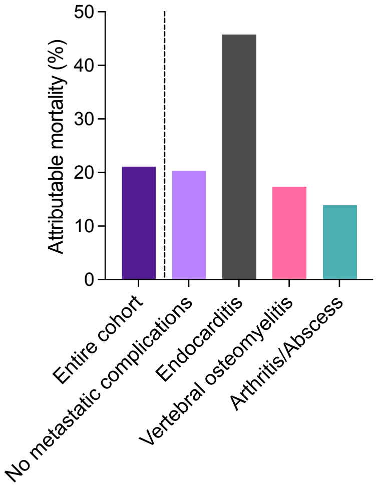

Supplementary Figure 8: Attributable in-hospital mortality stratified by metastatic complications. Patients were assigned to the three metastatic infection groups in a hierarchical manner: endocarditis, then vertebral osteomyelitis, then septic arthritis/metastatic abscesses.

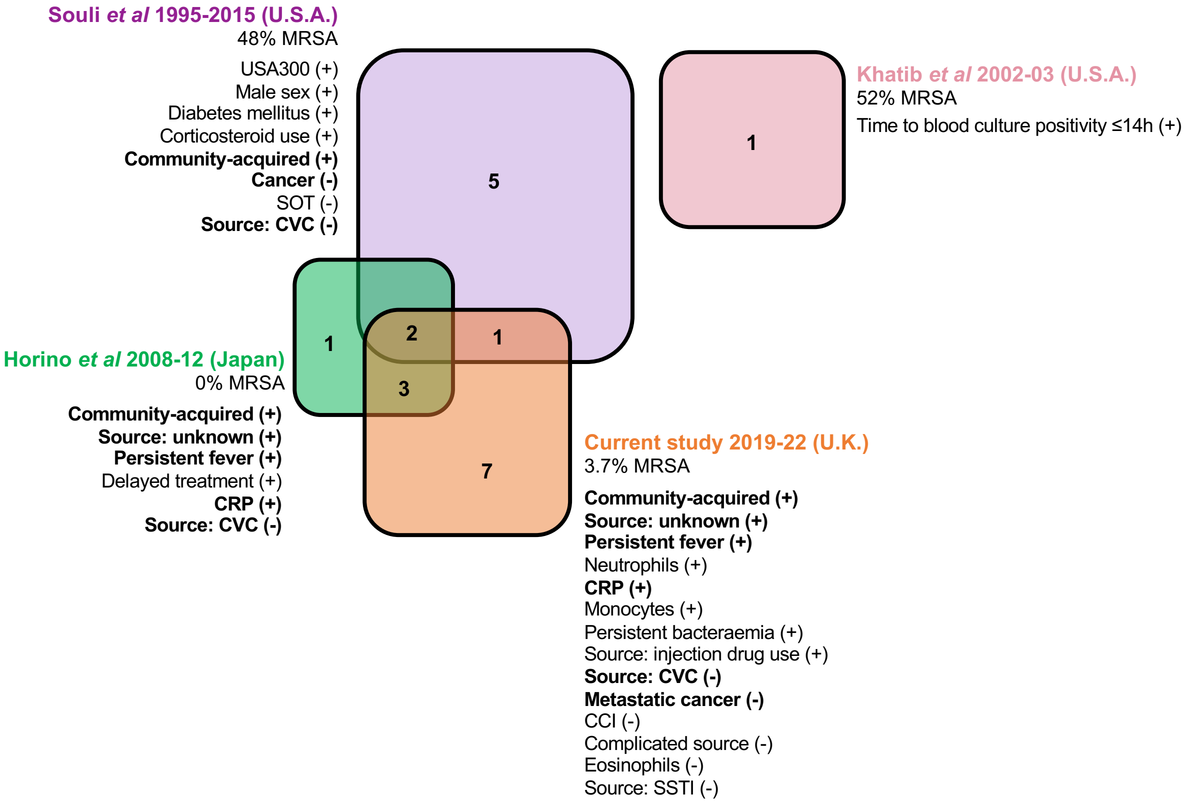

Supplementary Figure 9: Overlap in features of metastatic SAB between studies.
Variables shown in bold font overlap between ≥2 of the studies. (+) indicates a positive association and (-) indicates a negative association with presence of metastatic complications. Studies are cited in main manuscript.
